# Chronic Intermittent Hypoxia Alters Local Respiratory Circuit Function at the Level of the preBötzinger Complex

**DOI:** 10.3389/fnins.2016.00004

**Published:** 2016-02-04

**Authors:** Alfredo J. Garcia, Sebastien Zanella, Tatiana Dashevskiy, Shakil A. Khan, Maggie A. Khuu, Nanduri R. Prabhakar, Jan-Marino Ramirez

**Affiliations:** ^1^Center for Integrative Brain Research, Seattle Children's Research InstituteSeattle, WA, USA; ^2^Institute for Integrative Physiology, The University of ChicagoChicago, IL, USA; ^3^Departments of Neurological Surgery and Pediatrics, University of WashingtonSeattle, WA, USA

**Keywords:** preBötzinger complex, intermittent hypoxia, oxidative stress, obstructive sleep apnea, apneas of prematurity

## Abstract

Chronic intermittent hypoxia (CIH) is a common state experienced in several breathing disorders, including obstructive sleep apnea (OSA) and apneas of prematurity. Unraveling how CIH affects the CNS, and in turn how the CNS contributes to apneas is perhaps the most challenging task. The preBötzinger complex (preBötC) is a pre-motor respiratory network critical for inspiratory rhythm generation. Here, we test the hypothesis that CIH increases irregular output from the isolated preBötC, which can be mitigated by antioxidant treatment. Electrophysiological recordings from brainstem slices revealed that CIH enhanced burst-to-burst irregularity in period and/or amplitude. Irregularities represented a change in individual fidelity among preBötC neurons, and changed transmission from preBötC to the hypoglossal motor nucleus (XIIn), which resulted in increased transmission failure to XIIn. CIH increased the degree of lipid peroxidation in the preBötC and treatment with the antioxidant, 5,10,15,20-Tetrakis (1-methylpyridinium-4-yl)-21*H*,23*H*-porphyrin manganese(III) pentachloride (MnTMPyP), reduced CIH-mediated irregularities on the network rhythm and improved transmission of preBötC to the XIIn. These findings suggest that CIH promotes a pro-oxidant state that destabilizes rhythmogenesis originating from the preBötC and changes the local rhythm generating circuit which in turn, can lead to intermittent transmission failure to the XIIn. We propose that these CIH-mediated effects represent a part of the central mechanism that may perpetuate apneas and respiratory instability, which are hallmark traits in several dysautonomic conditions.

## Introduction

Every year thousands of patients succumb to the detrimental effects of hypoxia on the nervous system. Hypoxic conditions can be caused by stroke, emphysema, sleep disordered breathing, and extreme altitude. Of particular significance is the exposure to chronic intermittent hypoxia, which can result in detrimental consequences at multiple levels. Chronic intermittent hypoxia is the cardinal trait of obstructive sleep apnea (OSA; Malhotra and White, 2002; Young et al., 2002; Dempsey et al., 2010; Ramirez et al., 2013; Tan et al., 2013) and central apneas (Dempsey et al., 2010), including apnea of prematurity (Di Fiore et al., 2013). These disorders are associated with numerous neuronal consequences such as sleep fragmentation (Carreras et al., 2014) autonomic disturbances (Garcia et al., 2013a; Mansukhani et al., 2014), increased risk of stroke (Deak and Kirsch, 2014; Marshall et al., 2014), and cognitive decline (Gozal et al., 2012; Djonlagic et al., 2014). In apnea of prematurity adverse effects on neurodevelopmental outcome are common (Martin et al., 2011; Di Fiore et al., 2013). Interestingly, these alterations may occur with a large degree of individual variation (Lurie, 2011), which makes understanding the neuronal consequences of intermittent hypoxia very challenging.

While it is generally agreed that central apneas and apneas of prematurity have a central neuronal basis, it must be emphasized that OSA also has a significant neurobiological basis. OSA results from the temporary cessation in hypoglossal activity that triggers pharyngeal collapse (Remmers et al., 1978; Ramirez et al., 2013). Many factors contribute to the cessation of hypoglossal activity (White and Younes, 2012), including, for example, changes in motoneuronal output (Horner, 2007), peripheral sensory input (Prabhakar et al., 2007), arousal threshold (Chamberlin, 2013), and neuromodulation (Funk et al., 2011). However, each of these factors alone does not easily explain the intermittent loss of neuronal activity in the hypoglossal motor system during inspiration, the key event underlying the airway collapse in OSA (Ramirez et al., 2013). It is known that CIH has specific effects on the central neuronal network underlying breathing. For example, changes in postinspiratory activity within the Bötzinger complex, and respiratory modulated rostral ventrolateral medulla (RVLM) presympathetic neurons seem to be responsible for alterations in respiratory sympathetic interaction that could contribute to hypertension (Moraes et al., 2013; Molkov et al., 2014). But these studies do not address a potential link between CIH and hypoglossal activity. In the present study we demonstrate that CIH has a detrimental effect on neuronal network function in the preBötzinger complex (preBötC). This brainstem network is critical for breathing (Tan et al., 2008; Ramirez, 2011; Schwarzacher et al., 2011) and seems to contribute to several inspiratory rhythms, including eupnea, sighs and gasping (Lieske et al., 2000; St-John et al., 2007; Tan et al., 2008; Koch et al., 2013). Here, we test the hypothesis that CIH increases irregular output from the isolated preBötC, which can be mitigated by antioxidant treatment. We report that CIH alters not only this respiratory network, but also changes the input-output relationship between preBötC and XIIn leading to intermittent transmission failure to the hypoglossal motor nucleus. Furthermore, we find that CIH increases the degree of lipid peroxidation within the preBötC and that transmission from preBötC to XIIn can be protected by antioxidant treatment during CIH. We propose that the effects of CIH on the preBötC may contribute to the apneic events and the large spectrum of phenotypes known to exist among individuals who suffer from untreated OSA.

## Methods

### Ethics statement

Experiments were conducted using CD1 mice and protocols were approved by Seattle Children's Research Institute Animal Care and Use Committee in accordance with the National Institutes of Health guidelines. All animal subjects were housed at 21°C and in a 12 h/12 h light cycle where the light phase was from 07:00 to 19:00. Animals had access to food and water ad libum.

### Exposure to CIH

Neonatal mice (beginning from postnatal day 0 to 2) and their dam were exposed to a CIH paradigm similar to that described by Peng et al. (2004). To reduce the potential effects of maternal stress, litters exposed to CIH were culled down to a total eight pups prior to CIH exposure while control litters were left unculled (unculled litter size = 12–16). In a subset of animals, we compared the growth by the end of exposure to CIH or after 10 days left unexposed. The mass of control subjects [6.58 ± 0.29 g (*n* = 11)] was not different from that of CIH exposed subjects [7.07 ± 0.38 g (*n* = 15), *P* = 0.33; *F* = 2.24, *P* = 0.18]. The CIH paradigm was executed during the light cycle and lasted for 8 h/day (i.e., 80 intermittent hypoxia cycles/day) for 10 consecutive days. A single hypoxic cycle was achieved by flowing 100% N_2_ into the chamber for approximately 60 s that created a hypoxic environment where the nadir O_2_ chamber value reached 4–5% O_2_ (for 5–7 s). The hypoxic phase was immediately followed by an air break (300 s). The air break was achieved by flushing the chamber with air (~21% O_2_) restoring a normoxic state (18–20%) within 60 s following hypoxia. Environmental CO_2_ did not rise greater than 0.02% during any phase. Both pups and their dam were exposed to the CIH paradigm. In a subset of experiments, pups exposed to CIH were treated daily with the cell-permeable superoxide anion scavenger, 5,10,15,20-Tetrakis(1-methylpyridinium-4-yl)-21*H*,23*H*-porphyrin manganese(III) pentachloride, (MnTMPyP; 25 mg/kg daily; route: *i.p*.) throughout the entirety of exposure to CIH or for 10 days without CIH.

CD1 pups (*n* = 7) that received MnTMPyP treatment over the course of 10 days (from p1 to p10) without CIH exposure had similar masses to both control and CIH (9.53 ± 0.72 g at p10). Electrophysiological experiments were conducted where both preBötC and XIIn were simultaneously recorded (*n* = 3). The irregularity score of amplitude was 0.11 ± 0.03 and the irregularity score of period was 0.32 ± 0.08 in the preBötC and 100% transmission was observed between preBötC and XIIn (*data not shown*).

### Brain slice preparation and electrophysiological recordings

Transverse brainstem slices containing the preBötC (Hill et al., 2011; Garcia et al., 2013b; Zanella et al., 2014) were prepared from either CIH-exposed mice (12–48 h after the end of the CIH paradigm) or naïve CD1 mice (postnatal day 10–12). In brief, the isolated brainstem was glued to an agar block (dorsal face to agar) with the rostral face up and submerged in artificial cerebrospinal fluid (aCSF, ~4°C) equilibrated with Carbogen (95% O_2_, 5% CO_2_). Serial cuts were made through the brainstem until the appearance of anatomical landmarks such as XIIn and inferior olive. A single slice (550–600 μm) was retained containing the preBötC and XIIn.

The composition of aCSF was (in mM): 118 NaCl, 3.0 KCl, 25 NaHCO_3_, 1 NaH_2_PO_4_, 1.0 MgCl_2_, 1.5 CaCl_2_, 30 D-glucose. The aCSF had an osmolarity of 305–312 mOSM and a pH of 7.40–7.45 when equilibrated with gas mixtures containing 5% CO_2_ at ambient pressure. Rhythmic activity from the preBötC was induced by raising extracellular KCl to 8.0 mM. Control oxygen conditions were made by equilibrating aCSF with carbogen (95% O_2_, 5% CO_2_) while hypoxic conditions were made by aerating with 95% N_2_, 5% CO_2_. Despite the equilibration of aCSF with 0% O_2_, hypoxic media contained some O_2_ (Hill et al., 2011).

Extracellular population activity was recorded with glass suction pipettes (tip resistance <1 MΩ) filled with aCSF and were positioned over the ventral respiratory column containing the preBötC or the XIIn. Recorded signals were amplified 10,000X, filtered (low pass, 1.5 kHz; high pass, 250 Hz), rectified, and integrated using an electronic filter. In some cases, population recordings from two preBötC brainstem slices were recorded simultaneously within a single recording chamber. In situations where two slices were used, the slices were positioned in a staggered arrangement such that media flow was not obstructed for either preparation.

Intracellular recordings were made from putative inspiratory neurons of the preBötC using a multiclamp amplifier (Molecular Devices, Sunnyvale, CA). We defined the putative inspiratory preBötC neurons as neurons that received excitatory synaptic input in phase with the network rhythm—independent of degree of action potentials generated per cycle. These recordings were made in current clamp configuration using the blind patch clamp approach. Recordings were made using borosilicate glass recording electrodes (4–6 MΩ that were pulled using a P-97 Flaming/Brown micropipette puller (Sutter Instrument Co., Novato, CA) and filled with intracellular patch electrode solution containing (in mM): 140 potassium gluconate, 1 CaCl_2_, 10 EGTA, 2 MgCl_2_, 4 Na_2_ATP, and 10 Hepes (pH 7.2). The calculated junction potential of −12 mV was subtracted posthoc from membrane potential.

Both extracellular and intracellular recordings were acquired in pCLAMP software (Molecular Devices, Sunnyvale, CA) and were analyzed posthoc in Clampfit software (version 10.2).

### Lipid peroxidation

The degree of lipid peroxidation was determined by measuring malondialdehyde (MDA) levels in sectioned tissue samples containing either the preBötC or XIIn. Each sample n contained tissue pooled from two subjects either from the control or the CIH group. MDA levels were assayed as previously described (Garcia et al., 2011; Raghuraman et al., 2011) and expressed as nanomoles per milligram of protein.

### Statistical analysis

Short term variability of a given metric was quantitatively described by the irregularity score (Zanella et al., 2014). Kernel density estimation was conducted and plotted using Matlab. Comparisons between two groups were made using Unpaired Student's *t*-test with a Welch's correction to avoid assuming equal variances between groups using Graphpad Prism (Graphpad Software Inc., La Jolla CA). Unless otherwise stated, comparisons between two groups were plotted using box-whisker plots where the error bars represent the maximum and minimum values of the data. Numerical data reported were expressed as the mean ± standard error of the mean. Linear regression analysis was used to describe the fidelity of an individual neuron to the respective population rhythm.

Two and three-dimensional graphs of kernel density functions were calculated and plotted using Matlab. Univariate kernel density estimates the probability density function of a single trait or variable for a given data set. We also used bivariate kernel density (KS_2D_) to give a bivariate estimation of the probability density function of 2-dimensional data set. KS_2D_ was defined as:

$$\begin{array}{l} \text{K}{\text{S}}_{2\text{D}}\left(\text{x},\text{y}\right)=\text{KS}\left({\text{x}}_{\text{i},\ldots \text{n}}\right)\text{ x KS}\left({\text{y}}_{\text{i},\ldots \text{n}}\right), \end{array}$$

where KS(x_i,…n_) and KS(y_*i*,…n_) are a univariate kernel density functions of two independent data sets such as (1) irregularity scores of amplitude (x) and period (y) where n is sample size.

The ratio between the integrated burst areas generated in the hypoglossal nucleus (XII) and preBötC were used to examine the input-output (I/O) relationship between the pre-motor network (i.e., preBötC) and the motor pool (i.e., XIIn). We refer to this ratio as the I/O ratio, which as defined by the following equation:

$$\begin{array}{l} \text{I}∕\text{O }{\text{ratio}}_{\text{n}}=\int \text{B}{\text{A}}_{\text{XIIn}}∕\int \text{B}{\text{A}}_{\text{preBötCn}} \end{array}$$

where ∫BA_XIIn_ is the integrated burst area of XII of the n^th^ cycle and ∫BA_preBötCn_ is the corresponding integrated burst area of preBötC of the n^th^ cycle. In cases where a preBötC burst was detected but no corresponding burst was detected in XIIn, the integrated burst area of XIIn was assigned a zero value.

The standard deviation perpendicular (SD1) to the identity line and the standard deviation scattered along the identity line (SD2) were used to quantify the degree of dispersion in Poincare plots. SD1 described short term variability while SD2 described long term variability (Brennan et al., 2001).

## Results

### CIH exposure results in increased variability in respiratory population activity

**Figure 1:**
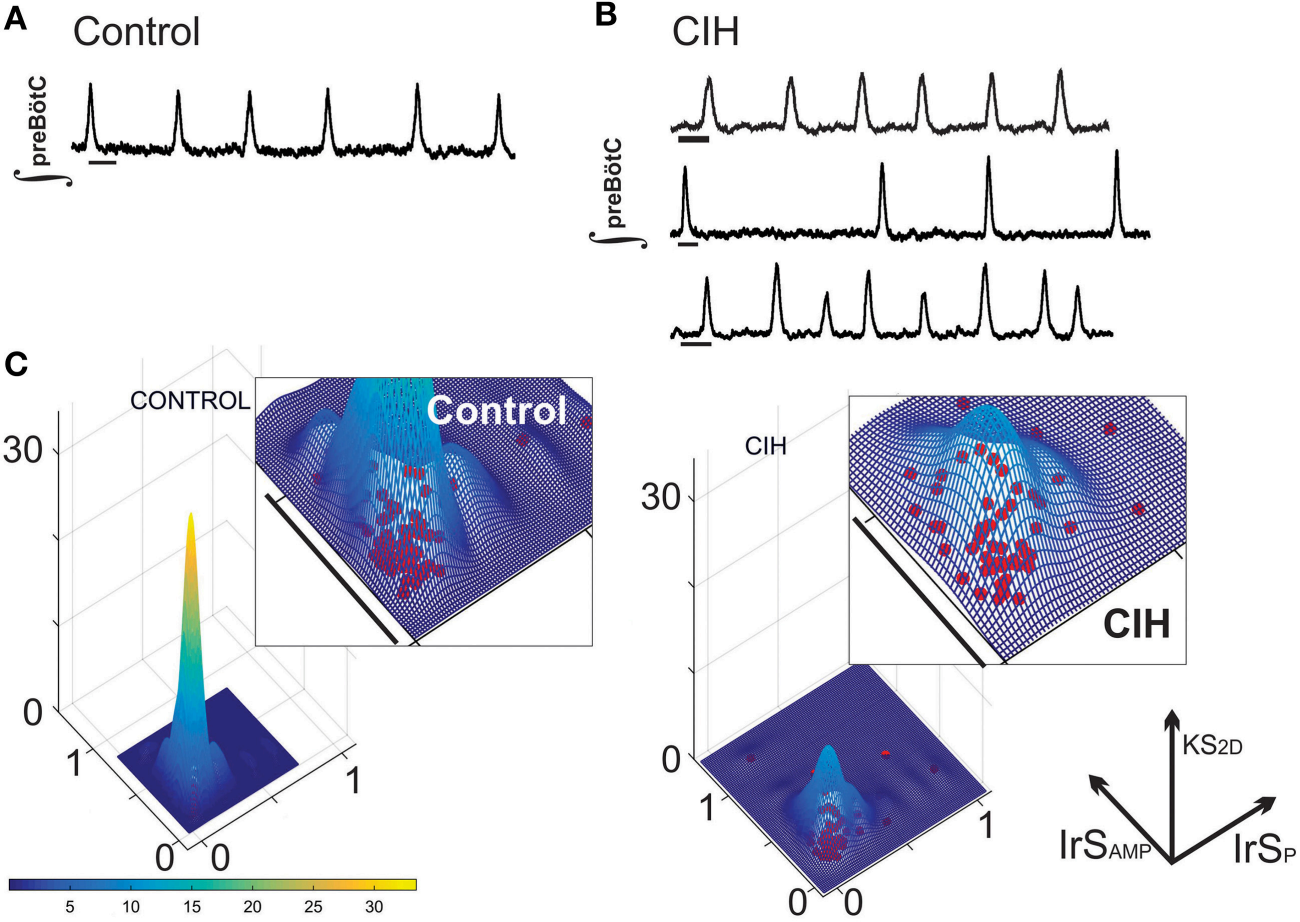
**CIH disperses short-term regularity of rhythm generation. (A)** Representative integrated trace of the network preBötC rhythm (∫) from a control slice where short-term (i.e., burst-to-burst) variability is regular. Scale Bar: 2 s. **(B)** While some preBötC rhythms from subjects exposed to CIH were indistinguishable (*top*), many rhythms exhibited obvious burst-to-burst variability in period (*middle*), and/or amplitude (*bottom*). Scale Bar: 2 s. **(C)** Plotting the kernel density estimation functions of the IrS_Amp_ and IrS_P_ for CIH and control groups reveals that CIH disperses the distribution of composite short-term variability compared to control rhythm.

Extracellular population recordings were made from the preBötC of control (*n* = 57) and CIH-exposed (*n* = 58) subjects. Although, all mice had the same genetic background (CD-1), CIH exposure resulted in an unexpected increase in individual variability of period and amplitude of respiratory activity. The central respiratory activity of some CIH-exposed subjects was indistinguishable from control (Figure 1A), but many CIH exposed individuals exhibited central respiratory rhythmic activities that were markedly more irregular in the amplitude and/or period of respiratory population activity (Figure 1B). To quantify these observations, we compared the irregularity score of amplitude (IrS_Amp_) and the irregularity score of period (IrS_P_) between rhythmic population activities of CIH exposed and control animals. Both the IrS_AMP_ (control: 0.17 ± 0.01, CIH: 0.24 ± 0.02; *P* = 0.004) and the IrS_P_ (control: 0.25 ± 0.01, CIH: 0.33 ± 0.02; *P* = 0.003) were greater in CIH treated groups. In agreement with our qualitative observations, the variance between the two groups was different for both IrS_P_ (F-ratio = 3.45, P_Ftest_ < 0.0001) and IrS_Amp_ (F-ratio = 2.11, P_Ftest_ < 0.006). This finding was readily demonstrated in the KS_2D_ of IrS_AMP_ and IrS_P_ of individual rhythms and indicates that CIH dispersed the tight distribution of composite burst-to-burst regularity of the preBötC rhythm that was characteristic for control animals (Figure 1C).

### CIH causes intermittent failure in the transmission from the preBötC to the hypoglossal motor pool

**Figure 2:**
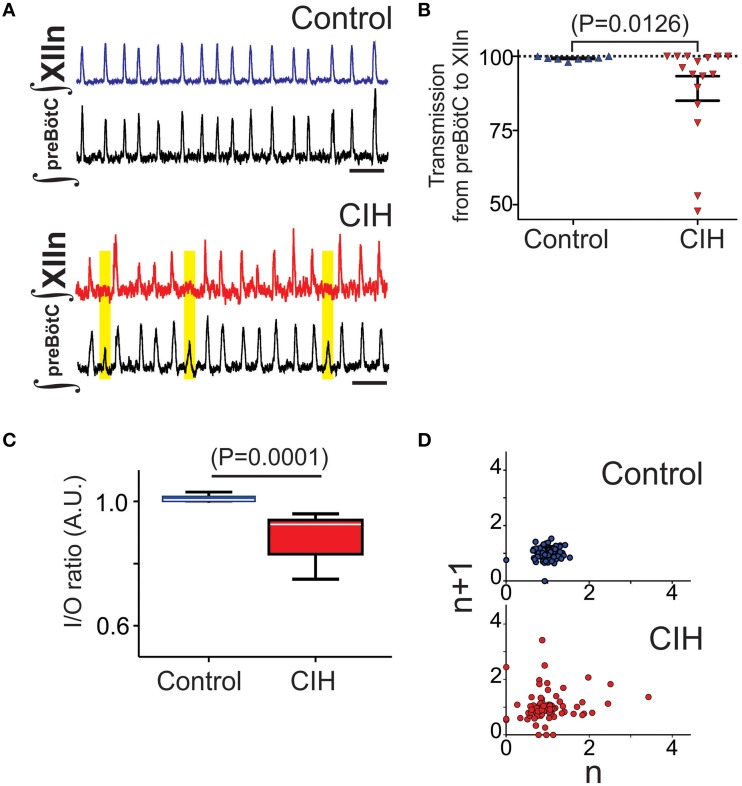
**CIH causes a mismatch between preBötC network bursting and the XIIn and disrupts the typically tight input-output relationship. (A)** Paired recordings of integrated preBötC and XIIn network rhythms from an individual control slice demonstrating the typical one-to-one burst relationship between premotor rhythm generator and the motor nucleus (*top*). Following CIH, network activity from the preBötC intermittently fails to produce activity in the XIIn (*bottom*). Yellow boxes highlight mismatch. Scale Bar: 10 s. **(B)** Transmission from preBötC to XIIn is reduced following CIH (n_CIH_ = 16, n_control_ = 10). Furthermore, the CIH group exhibited a larger variance in rate of intermittent transmission failure compared to the control group (F-ratio = 690, *P* < 0.001). **(C)** When transmission from preBötC to XIIn was <90% following CIH, the I/O ratio revealed that CIH disrupts the I/O relationship between the premotor rhythm generator and the motor pool normally observed in control (F-ratio = 51.63, *P* < 0.0001). **(D)** Representative poincare maps of the I/O ratios illustrating that CIH also disrupted the normal predictability of I/O relationship. The dispersion in both SD1 and SD2 was different between control and CIH (*see text*).

Simultaneous extracellular recordings of the population rhythms from the preBötC and the corresponding XIIn were recorded in brainstem slices from CIH and control subjects. Intermittent failure in transmission of the preBötC rhythm to the hypoglossal network was evident in the CIH group and contrasted the reliability observed in the control group (Figure 2A). In networks with increased amplitude irregularity after CIH exposure, failed transmission to XIIn occurred during network bursts that were characterized by small amplitudes (Figure 2A, yellow boxes). Transmission from the preBötC to XIIn was lower following CIH (Figure 2B; *n* = 10 CON, *n* = 16 CIH; *P* = 0.01, F-ratio = 690, *P* < 0.0001). Comparing the I/O ratios from networks exposed to CIH with transmission rates <90% (*n* = 5) to the control group revealed a significant, yet variable, reduction in the input-output relationship between preBötC and XIIn (Figure 2C, *P* = 0.01; F-ratio = 41.63, *P* < 0.00001). Poincare plots of the I/O ratio revealed that CIH exposure led to erratic transmission of the preBötC rhythm to XIIn (Figure 2D). Quantitative analysis of short-term variability (SD1: Control = 0.24 ± 0.04, CIH = 0.44 ± 0.08, *P* = 0.02; F-ratio = 2.64 *P* = 0.208) and long term variability (SD2: Control = 0.31 ± 0.04, CIH = 0.54 ± 0.09, *P* = 0.014; F-ratio = 3.05 *P* = 0.16) in the Poincare plots were different following CIH. Together these data suggests that CIH changed the I/O relationship and this change correlated to the increased irregularity of preBötC amplitude.

### CIH alters the coupling between the activity of individual neurons and the network population rhythm

**Figure 3:**
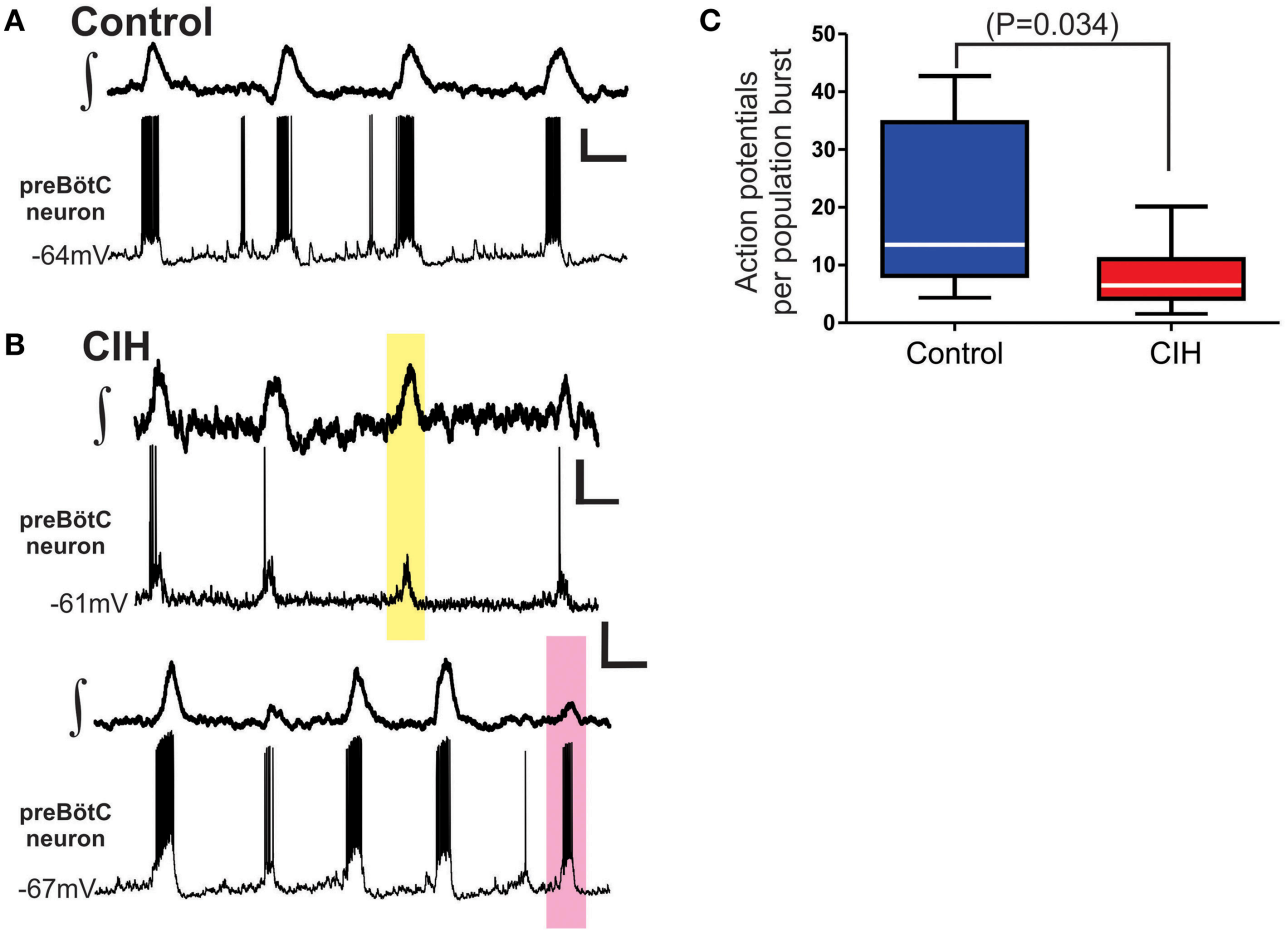
**CIH alters neuronal excitability coupled to the network rhythm. (A)** Representative traces paired recordings of the integrated preBötC network rhythm (∫,*top*) and membrane potential (V_m_) of an individual preBötC neuron participating with the network (*bottom*) from a control slice. **(B)** Representative traces of paired recordings from two individuals slices illustrating the change in firing pattern during network activity and fidelity of individual preBötC neurons relative to the network rhythm following CIH. The yellow box highlights the lack of action potential generation from the preBötC neuron during network burst (*top pair*). The pink box highlights robust action potential generation from a preBötC neuron occurring during a smaller subnetwork network burst (*bottom pair*). Vertical Scale Bars: 10 mV; Horizontal Scale Bars: 500 ms. **(C)** Summarized data shows that, in aggregate, number of action potentials generated by individual preBötC neurons during the network burst is reduced following CIH.

To further understand the impact of CIH on the preBötC, we recorded the activity of individual preBötC neurons. Exposure to CIH changed the reliability of action potential generation during the network burst (Figure 3A), which resulted in fewer action potentials generated during the population burst in network obtained from animals that were exposed to CIH (Figures 3B,C; control: 20 ± 5, *n* = 10; CIH: 8 ± 1, *n* = 16; *P* = 0.034, F-ratio = 7.84, *P* = 0.008). This raised the question whether CIH changed the fidelity of activity between an individual neuron and the network population rhythm.

**Figure 4:**
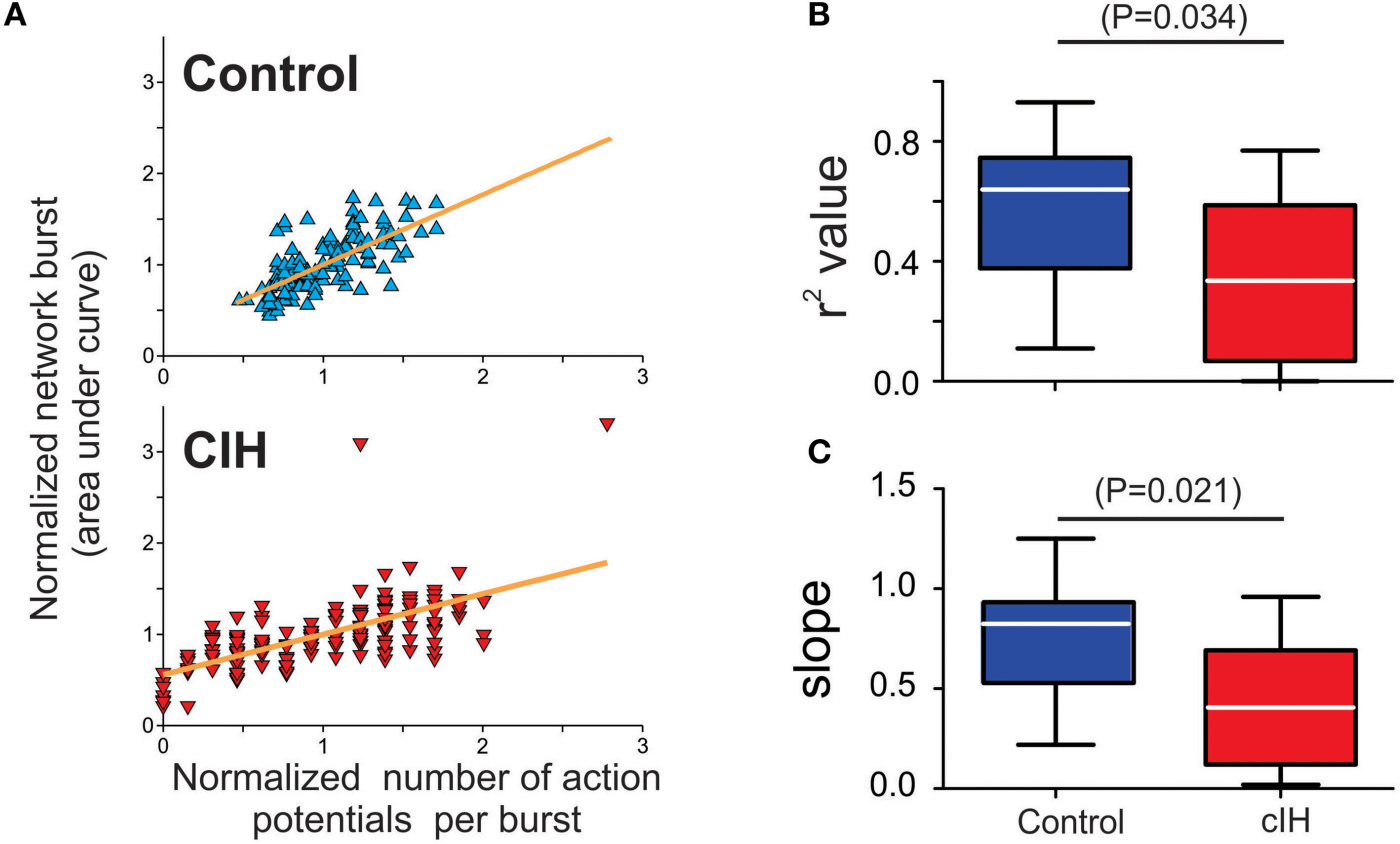
**CIH disrupts the predictable linear relationship between action potential generation of individual preBötC neurons and network activity. (A)** Individual examples of linear regression analysis run on a single neuron and the respective network burst in slices from a control and a CIH rhythm. Because the CIH reduced the absolute output from a given preBötC neuron, it was important to normalize the number of action potentials generated from a given preBötC neuron to the mean number of action potential produced during the network burst to accurately assess whether CIH changed the behavior of an individual neuron relative the corresponding network burst. **(B)** The coefficient of determination value (r^2^) is larger in the control group compared to CIH group **(C)** Similar to r^2^, the slope value is larger in the control group compared to the CIH group.

To account for a potential masking effect due to the differences in absolute output between control and CIH exposed neurons, we examined the relationship between normalized output of an individual neuron to that of the corresponding network burst. This analysis revealed that the output range an individual neuron (i.e., the number of action potentials generated during a network burst) expands following CIH when compared to control neurons (Figure 4A). Specifically, the number of action potentials generated by a single neuron is often attenuated and even absent despite the generation of network activity. Linear regression analysis revealed that both r^2^-values (control: 0.58 ± 0.08, CIH: 0.35 ±.06; *P* = 0.034; F-ratio = 1.11, *P* = 0.90) and fitted slope values (control: 0.75 ± 0.10, CIH: 0.44 ± 0.08; *P* = 0.021; F-ratio = 1.38, *P* = 0.99) are reduced following CIH (Figures 4B,C). The smaller r^2^-value in the CIH group indicated that the number of action potentials are weaker predictors of network activity following CIH. Secondly, the smaller slope also suggested that changes in number of action potentials from these neurons have a smaller impact on network activity following CIH. Thus, the effects of CIH at the single cell level extend beyond a simple reduction of overall neuronal excitability, but rather appears to involve a change in the predictive fidelity of individuals in participating in the network rhythm.

### Antioxidant supplementation mitigates the effects of CIH on rhythmogenesis in the isolated respiratory circuit

**Figure 5:**
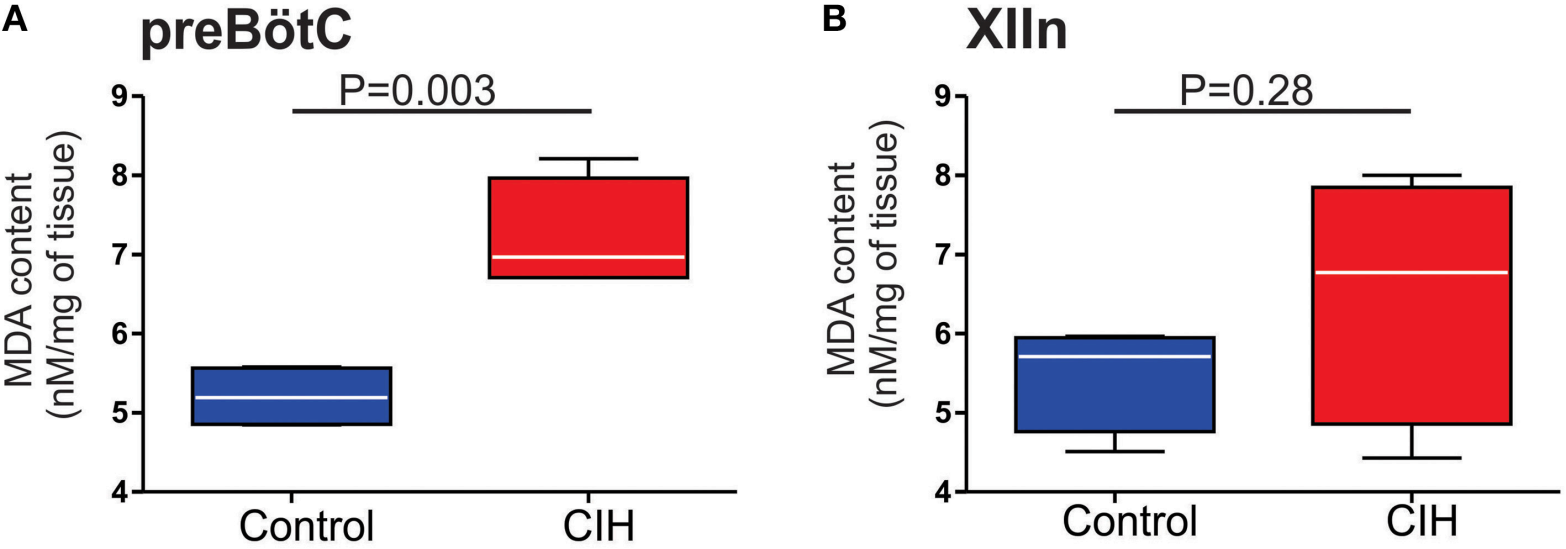
**Effect of CIH on the degree of lipid peroxidation in the preBötC and XIIn**. Summarized MDA content measured in tissue homogenates from **(A)** the preBötC (*n* = 4 samples) and **(B)** XIIn (*n* = 4 samples).

**Figure 6:**
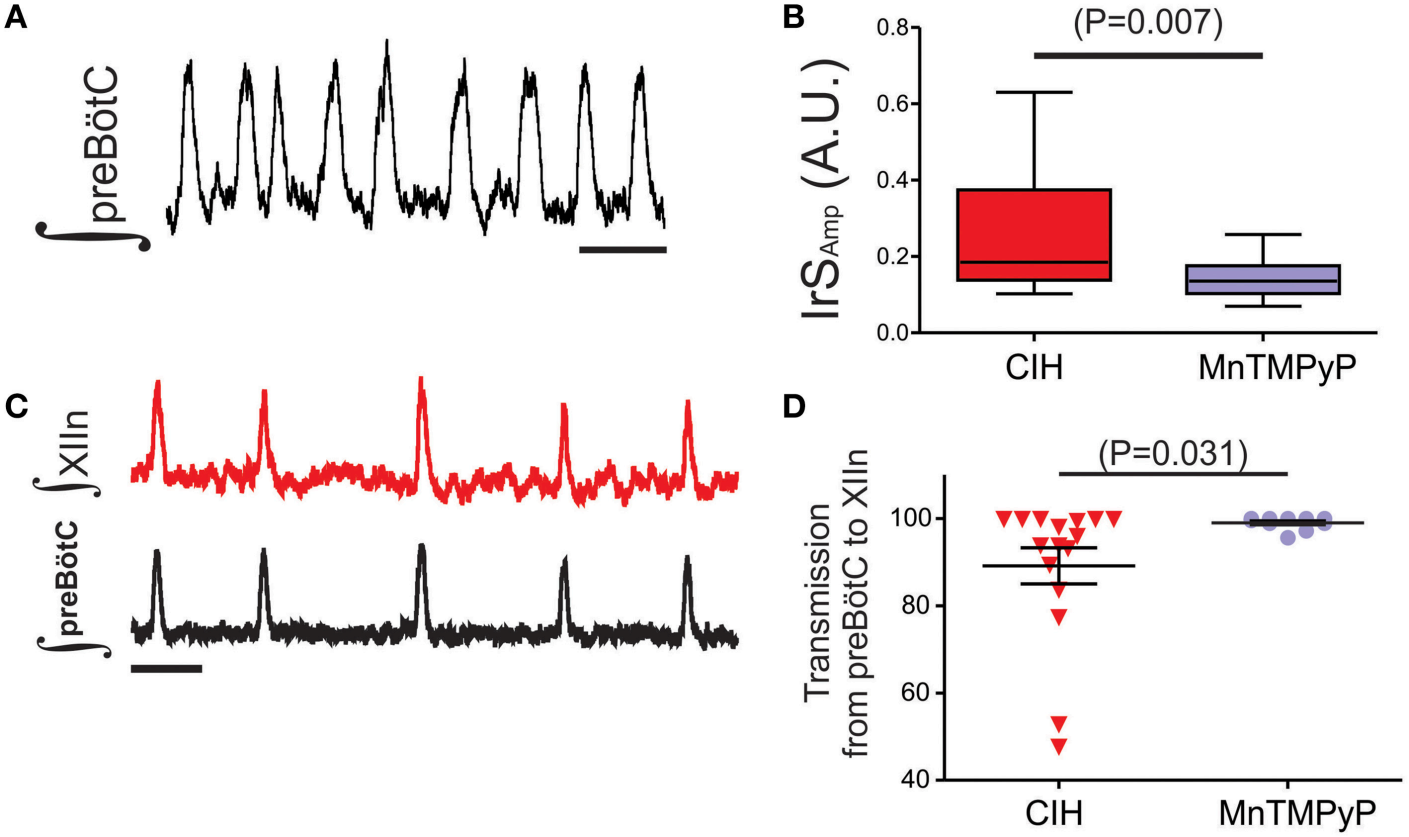
**MnTMPyP treatment reduces CIH-mediated amplitude irregularities within the preBötC and preserves rhythm generation in Xlln. (A)** Individual example of rhythmogenesis from the preBötC following MnTMPyP treatment during CIH. Scale Bar: 4 s. **(B)** Summarized comparison of IrS_AMP_ illustrating the difference between preBötC rhythms exposed to CIH alone (*n* = 20) or treated with MnTMPyP during CIH (*n* = 18). **(C)** A representative trace from a subset of experiments (*n* = 10) where simultaneous recordings from the preBötC and XIIn following MnTMPyP treatment during CIH were made. Scale Bar: 4 s. **(D)** As summarized in the plot, transmission from preBötC to XIIn is restored following MnTMPyP treatment during CIH when compared the CIH group.

The augmenting effects of CIH on the output from the carotid bodies of the peripheral nervous system can be mitigated with antioxidant treatment and suggested that CIH promotes pro-oxidant state which affects physiology in the nervous system (Pawar et al., 2009). Furthermore, our previous work in the preBötC demonstrated that exogenous reactive oxygen species and oxidative stress significantly affects rhythmogenesis from the preBötC (Garcia et al., 2011). Thus, to determine whether CIH affected the pro-oxidant state within the preBötC, we measured the amount of MDA, a common end product of lipid peroxidation resulting from oxidative stress. In the preBötC, amount of MDA was larger in control (5.21 ± 0.20 nM per mg; *n* = 4) compared to CIH (7.22 ± 0.35 nM per mg; *n* = 4). While this difference in the amount of MDA was significant in the preBötC (Figure 5A; *P* = 0.003; *F* = 3.14, *P* = 0.37), no differences were observed in MDA from XIIn (Figure 5B; control: 5.48 ± 0.34 nM/mg, *n* = 4 vs. CIH 6.50 ± 0.79 nM/mg, *n* = 4; *P* = 0.28). Based on these observations, we sought to determine whether the provision of the antioxidant, MnTMPyP (25 mg/kg), affected the outcome of CIH on the isolated rhythmic network. Although MnTMPyP treatment did not appear to affect CIH-mediated period irregularities (i.e., in IrS_P_ was not different between the CIH and CIH with MnTMPyP, *P* = 0.24; F-ratio = 1.90, *P* = 0.28), the antioxidant appeared to prevent amplitude irregularities within the preBötC (Figure 6A; *n* = 12). IrS_Amp_ between rhythms from subjects exposed CIH alone and rhythms from subjects receiving MnTMPyP during CIH was different (Figure 6B; *P* = 0.018; F-ratio = 6.72, *P* = 0.002). MnTMPyP treatment during CIH also appeared to preserve transmission of preBötC the rhythm to XIIn (*n* = 10; Figure 6C). This was confirmed by the difference in transmission rates between the CIH and the antioxidant group (Figure 6D; CIH: 89.15 ± 4.12%; MnTMPyP: 99.02 ± 0.471%; *P* = 0.03; F-ratio = 122.5, *P* < 0.001).

## Discussion

This study demonstrates that CIH has a major impact on the central mechanisms of rhythm generation within the preBötC, the putative site for the generation of inspiratory activity (Smith et al., 1991; Ramirez, 2011). Isolated respiratory networks from subjects exposed to CIH show a wide distribution of composite irregularity not observed in the control group. Network irregularities could be traced to individual neurons and can be accompanied by compromised transmission to the Xlln, which in its extreme, resulted in brief pauses in rhythmic hypoglossal motor activity. Moreover, antioxidant supplementation reduced the impact of CIH on amplitude irregularity within the preBötC and restored transmission from the premotor network to XIIn. Together these findings provide a potential understanding into the central mechanisms that may perpetuate or contribute to respiratory disturbances associated with intermittent hypoxia.

In the context of cardiorespiratory control, previous studies have demonstrated that abdominal nerve and thoracic sympathetic nerve discharge changes in both normocapnia and hypercapnia following CIH (Abdala et al., 2009; Zoccal et al., 2009a,b). These observations suggest that a neurophysiological remodeling of central cardio-respiratory networks occurs in response to CIH. Indeed, a subset of RVLM presympathetic neurons receive enhanced excitatory synaptic inputs following CIH (Moraes et al., 2013). CIH also reduces the number of active glutamatergic synapses received by NTS neurons (Almado et al., 2012) and modulates both TrkB and BDNF protein expression in the nucleus (Moreau and Ciriello, 2015). Thus, these observations may assist in explaining central network contributions to CIH-mediated hyperactivity and hypertension (Molkov et al., 2014) but do not adequately answer how CIH may affect central networks with respect to respiratory control.

Our analyses suggest that CIH not only affected the preBötC but had significant effects throughout the local respiratory circuit. Specifically, the weakened and irregular input from the preBötC was accompanied by failed output at the level of XIIn, and when the rate of transmission failure was greater than 10%, significant changes in I/O relationship between the premotor network and the motor pool were also clearly evident. Thus, in addition to the effects at the level of the preBötC, CIH may also destabilize other portions of the local respiratory circuit, including premotor neurons that relay inspiratory drive from the preBötC to XIIn or even the motor pool itself. Such changes at any of these levels outside the preBötC may further amplify the unstable output from the premotor rhythm generator thus, exaggerating the effects observed at XIIn.

Multiarray recordings have revealed that each population burst is the result of a dynamic assembly of diverse neurons, rather than the outcome of ordered recruitment in which every cycle follows a deterministic activation order of similar neurons (Carroll and Ramirez, 2013; Carroll et al., 2013). We found that overall, fewer action potentials were generated from CIH exposed preBötC neurons during the network burst. Linear regression analysis also indicated that the fidelity of individual inspiratory neurons to a given respiratory cycle had changed following. These changes contribute to irregular and smaller network bursts, which typically failed to transmit to XIIn. Transmission failures to XIIn have previously been described in response to acute intermittent hypoxia (Zanella et al., 2014), and in conditions of reduced excitability at the level of the respiratory rhythm generator (Kam et al., 2013), or as a function of postnatal development (Ramirez et al., 1996). In all these cases preBötC activity was insufficient to reliably trigger bursts in the XIIn. But, the CIH induced alterations were fundamentally different since the chronically altered baseline state of the preBötC was not only characterized by amplitude, but also frequency irregularities. Increased individual variability was also characteristic for the CIH exposed network, which was not observed in networks from control animals.

We show that CIH increases lipid peroxidation in the preBötC. Moreover, antioxidant supplementation mitigates: (1) much of the CIH-mediated irregularities in rhythmogenesis; and (2) intermittent transmission failures of the preBötC rhythm to XIIn. The potential role of oxidative stress is reminiscent to the situation in the carotid bodies where the antioxidant, MnTMPyP, attenuates much of the CIH mediated oxidative stress and sensory long-term facilitation (Pawar et al., 2009). In the peripheral nervous system intermittent hypoxia mediated oxidative stress appears to originate from an imbalance of reactive oxygen species metabolism favoring the pro-oxidant state. Specifically, intermittent hypoxia appears to increase reactive oxygen species production from NADPH oxidase (Semenza and Prabhakar, 2015). Although the exact origin(s) by which oxidative stress may arise within the preBötC is not identified here, we previously demonstrated that hydrogen peroxide and oxidative stress significantly affects both the frequency and amplitude of rhythmogenesis from the preBötC and involved changes in both input resistance and excitability of synaptically isolated preBötC neurons (Garcia et al., 2011). Similarly, hydrogen peroxide appears to reduce the amount of synaptic input received by XIIn motoneurons (Nani et al., 2010).

Thus, disrupted synaptic transmission which leads to uncoupling between preBötC and XIIn caused by CIH is likely the result of a combination of effects caused by oxidative stress on the preBötC, premotor neurons outside the rhythm generator, and parahypoglossal neurons.

In addition to the effects that could be alleviated with antioxidant supplementation, we observed a large degree of variability among individual networks from subjects with the same genetic background and identical experimental paradigm. This individual variability was readily reflected in the greater standard deviation and F-ratios >1 in IrS_AMP_ and transmission from preBötC to XIIn. This is particularly interesting given the fact that the severity of respiratory disturbance varies among individuals who suffer from both apneas of prematurity and sleep disordered breathing, such as OSA (Edwards et al., 2014; Terrill et al., 2015), but is uncommon in experimental reports studying the effects of CIH. The individual variability we observed in the respiratory network may lend itself as a potential central mechanism that contributes to the diversity of presentation in respiratory disorders. However, we recognize that a high degree of overlap and interaction exists among central networks which regulate cardio-respiratory control. A high degree of overlap and interaction exists among central networks which regulate cardio-respiratory control. Thus, future studies are required to examine how greater levels of physiological organization may augment or even compensate for the effects we observed here. Work using models, such as the working heart brainstem or *in vivo* preparations, will be important next steps to obtaining a more complete understanding of our findings in the isolated preBötC.

An important caveat to our study is the potential influence of CIH on the postnatal development of the respiratory network. Although, we did not further explore this aspect, understanding the potential effects on neurodevelopment is of important clinical relevance for understanding the detrimental consequences of apnea of prematurity (Martin et al., 2011; Di Fiore et al., 2013) and also pediatric OSA (Lopes and Marcus, 2007; Hakim et al., 2015; Kaditis et al., 2016).

We believe our study identifies a novel mechanism that increases the likelihood for the airway collapse in OSA (Remmers et al., 1978; Horner, 1996; Bailey et al., 2006; Ramirez et al., 2013). To this end, it is well recognized that the effects of OSA may increase in severity when left untreated. Thus, our findings may indeed be an important mechanistic contributor to respiratory disturbances independent of developmental stage.

In conclusion, our study demonstrates that CIH has marked effects on the central respiratory network underpinning inspiratory rhythm generation, which could be mitigated by antioxidant supplementation. Because these CIH-mediated disturbances to premotor rhythmogenesis are accompanied by intermittent failures in motor output, these disturbances may act as a central mechanism that perpetuates and/or to contribute to the development respiratory instabilities in conditions associated with intermittent hypoxia.

## Author contributions

Conceived and designed the experiments: AG, SZ, TD, and JR. Performed the experiments: AG, SZ, TD, SK, and MK. Analyzed the data: AG, SZ, TD, and SK. Contributed reagents/materials/analysis tools: NP and JR. Wrote the paper: AG, SZ, TD, NP, and JR.

## Funding

This study was supported by National Institutes of Health Grants R01 HL107084-01 and P01-HL-090554-01.

### Conflict of interest statement

The authors declare that the research was conducted in the absence of any commercial or financial relationships that could be construed as a potential conflict of interest.
